# The molecular determinants for distinguishing between ubiquitin and NEDD8 by USP2

**DOI:** 10.1038/s41598-017-02322-x

**Published:** 2017-05-23

**Authors:** Yung-Cheng Shin, Jou-Han Chen, Shih-Chung Chang

**Affiliations:** 1 Department of Biochemical Science and Technology, College of Life Science, National Taiwan University, Taipei, Taiwan; 2 Center of Biotechnology, National Taiwan University, Taipei, Taiwan

## Abstract

Ubiquitin (Ub) shares the highest sequence identity with neuronal-precursor-cell-expressed developmentally downregulated protein-8 (NEDD8) in the Ub-like protein family. However, different enzyme systems are precisely employed for targeting Ub and NEDD8 to specific substrates. The molecular determinants for distinguishing between Ub and NEDD8 by Ub-specific peptidases (USPs) remain poorly characterized. By replacing the non-conserved residues of Ub with their NEDD8 equivalents by mutagenesis, and vice versa, we observed that the Ub^4K^, Ub^12E^, and Ub^14E^ mutants partially and the Ub^4K/12E/14E/72A^ mutant completely prevented their hydrolysis by USP2. The NEDD8^4F^ and NEDD8^14T^ mutants were slightly hydrolyzed by USP2; however, the NEDD8^12T/14T/72R^ and NEDD8^4F/12T/14T/72R^ mutants were accessible for hydrolysis by USP2, suggesting that Ub and NEDD8 residues 4, 12, 14, and 72 serve as the molecular determinants for specific recognition by USP2. We also demonstrated that the level of inhibition caused by Ub mutants with multiple mutation sites was not purely additive when compared with the single mutation results. Furthermore, USP2 was determined to bind to the N-terminus of Ub to form a stable interaction, after which it binds with the C-terminus of Ub to ensure substrate specificity. The same results were also discovered when Ub, Ub^4K/12E/14E/72A^, NEDD8, and NEDD8^4F/12T/14T/72R^ were incubated with USP21.

## Introduction

Ubiquitin (Ub), a highly conserved 76-amino-acid polypeptide, is conjugated to target proteins through a process called ubiquitination mediated by the enzyme cascades comprising Ub-activating enzyme (E1), Ub-conjugating enzymes (E2), and Ub ligase (E3). Many Ub-like proteins (Ubls) have been identified in cells that have high sequence similarity and structural correlation with Ub. In general, different Ubls are believed to share neither E1, E2, and E3 enzymes nor cellular functions^1, 2^. However, different studies have revealed that the autophagy-related protein 8 (ATG8) and ATG12 are activated by the same E1 enzyme ATG7^3^, and Ub and HLA-F adjacent transcript 10 (FAT10) are activated by Uba1/Ube1 and Uba6^4–6^. Ub and Ubls are initially synthesized as precursors, after which they undergo proteolytic processing by deubiquitinating enzymes^7^ to yield their mature forms with a C-terminal glycine as the site of substrate attachment^8^. Dynamic conjugation or de-conjugation by Ubls is critical for the control of numerous key cellular processes and is itself tightly regulated. Additionally, the modification of target proteins by different Ubls may have distinct biological consequences^1, 2, 9–12^.

Among different Ubls, the neural-precursor-cell-expressed developmentally downregulated protein-8 (NEDD8)^13^ is the closest relative to Ub (58% sequence identity and 80% sequence similarity). NEDD8 can be conjugated to target substrates in a process that is similar to ubiquitination, called neddylation, which relies on its own E1 and E2 enzymes^14–16^. However, it has been shown that NEDD8 can be activated by Uba1/Ube1 upon increasing the ratio of free NEDD8 to Ub by overexpression in cells^17^, or under diverse stress conditions^18^, indicating that there is a cross-talk between these two pathways^19^. Moreover, human cells contain a highly specific protease, sentrin-specific protease 8 (SENP8), which has an exclusive preference for NEDD8 over Ub^20^ and cannot cleave Ub or small Ub-like modifier precursors^21^. Apart from SENP8, another best-characterized NEDD8 isopeptidase is the COP9 signalosome (CSN)^22^, which can deneddylate NEDD8-conjugated cullin-RING ubiquitin E3 ligases (CRLs)^23, 24^. Notably, unlike SENP8, CSN can deneddylate mononeddylated CUL1, but does not deconjugate hyper-neddylated CUL1^25^. Additionally, some proteases exhibit dual specificity for Ub and NEDD8 precursors, including USP21^26^, Ataxin-3^27^, UCH-L3^28, 29^, PfUCH54^30^, and Yuh1^31^.

By contrast, human Ub-specific peptidase (USP) 2 can function on Ub but not on NEDD8. Therefore, the mechanism through which SENP8 and USP2 exhibit this unique substrate specificity must be determined. In earlier studies, we^32^ and other groups^33, 34^ have discovered that residues at positions 51 and 72 of NEDD8 served as the molecular determinants for specific substrate recognition by SENP8. We have also demonstrated that the mutation of residues at positions 51 and 72 in Ub only partly inhibits Ub’s hydrolysis by USP2, indicating that USP2 requires additional substrate recognition sites. Thus, the molecular determinant for substrate differentiation between Ub and NEDD8 by USP2 remains to be determined.

The molecular structures of USP2^35^, HAUSP (as known as USP7)^36, 37^, USP14^38^, and USP21^39^ in complex with Ub have been characterized in several studies, revealing that different USPs share a common Fingers-Palm-Thumb catalytic domain architecture. However, the active site conformation of USPs can be misaligned or already well formed. Thus, the conformational changes of USPs triggered by Ub binding can be described by different models so that the enzyme activation mechanisms for different USPs are especially complicated and require further detailed characterization.

The 40-kDa catalytic core domain of HAUSP undergoes several dramatic conformational changes upon binding with Ub aldehyde (Ubal), a potent inhibitor of most USPs^40, 41^. Ubal forms a covalent complex with the catalytic cysteine of HAUSP, resulting in a root-mean-square deviation (RMSD) of 1.3 Å for 320 aligned Cα atoms between the free and Ubal-bound HAUSP structures^36^. Therefore, it is not surprising that free Ub does not form a stable complex with HAUSP and that high *K*_m_ values were observed in deubiquitination assays with Ub substrates^36^. However, the realignment of the active site of HAUSP can be induced by Ub binding, leading to the specific recognition and processing of Ub. Additionally, residues at the HAUSP-Ubal binding interface make crucial contributions to the binding of Ubal, including several hydrogen bonds and van der Waals interactions, that primarily involve the N-terminal residues of Ubal as well as a few amino acids in the middle stretch and at the C-terminus.

The 45-kDa catalytic domain of USP14 in isolation and in a complex with Ubal reveal that USP14 also has conserved three-domain architecture; however, the active site of free USP14 is already formed before substrate binding. Thus, the activation mechanism for USP14^38^ is quite different from that for HAUSP^36^. Although the substrate binding and activation mechanisms of HAUSP and USP14 are different, both mechanisms ensure appropriate catalytic activity and substrate specificity.

The conjugation of USP21 with a Ub-based suicide probe, Ub-C2Cl^42^, provides more insights on the substrate recognition mechanism of the USP protein family. USP21 largely conjugates with Ub-C2Cl; however, USP21 partially conjugates with UbR72A-C2Cl, indicating that Ub Arg-72 plays an essential role in binding with USP21^39^. However, an NEDD8 mutant was not able to bind to USP21 by simply mutating Ala-72 to Arg, suggesting that other differences between Ub and NEDD8 restrict USP21 activity toward NEDD8. Under this assumption, three residues (Phe-4, Thr-12, and Thr-14) in the N-terminal region of Ub, which interact with USP21, are proposed to play key roles in substrate-specific binding. The differences are well conserved between Ub and NEDD8 in different eukaryotes. The structural modelling of USP21-Ub^39^ and USP2-Ub complexes^35^ in which Ub has been replaced by the NEDD8 equivalent residues at positions 4, 12, and 14 results in steric clashes and charge repulsion between their interaction surfaces. Furthermore, Ye *et al*. reported that an NEDD8 suicide probe, in which four residues (positions 4, 12, 14, and 72) were changed to their Ub equivalents, was able to react with USP21^39^. Hence, both the C-terminal residue 72 and N-terminal residues 4, 12 and 14 of Ub contribute to the ability of USP21 to discriminate between Ub and NEDD8.

Investigating whether other USP proteins use similar mechanisms to distinguish between Ub and NEDD8 is critical; however, most USPs remain poorly characterized^43, 44^. The crystal structure of the USP2 catalytic core, comprising residues 259–605, in complex with Ub reveals that the Ub core (residues 1–71) binds into the Fingers-Palm-Thumb structural elements, whereas its five C-terminal residues (72–76) bind into a narrow channel and reach for the active site cysteine^35^. In fact, the different truncated forms of the Ub core (residues 1–71, 1–72, and 1–73) and the various short peptides of the C-terminus of Ub (residues 72–76 and 68–76) alone bind USP2 weakly^35^, suggesting that both interactions are required simultaneously for appropriate binding of Ub. However, it remains unknown whether the binding of USP2 to these two low-affinity sites is cooperative or purely additive. In addition, the molecular determinants for the discrimination between Ub and NEDD8 by USP2 remain elusive.

## Results

### The key residues of Ub recognized by USP2 for Ub-specific binding

**Figure 1:**
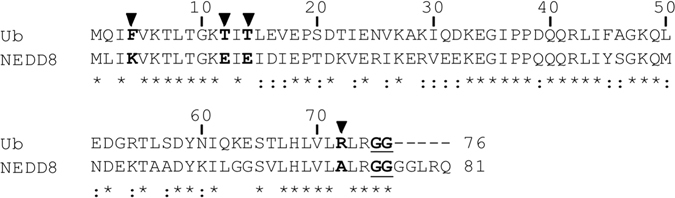
The sequence alignment of human Ub and NEDD8. The amino acid sequences of human Ub and NEDD8 were aligned using ClustalW. Identical residues were marked with asterisks. The non-conserved residues at positions 4, 12, 14, and 72 of Ub and NEDD8 were denoted in bold and marked with solid triangles, where the residues are phenylalanine, threonine, threonine and arginine in Ub and the equivalent residues are lysine, glutamate, glutamate and alanine in NEDD8. The C-terminal di-glycine motif was underlined and denoted in bold.

**Figure 2:**
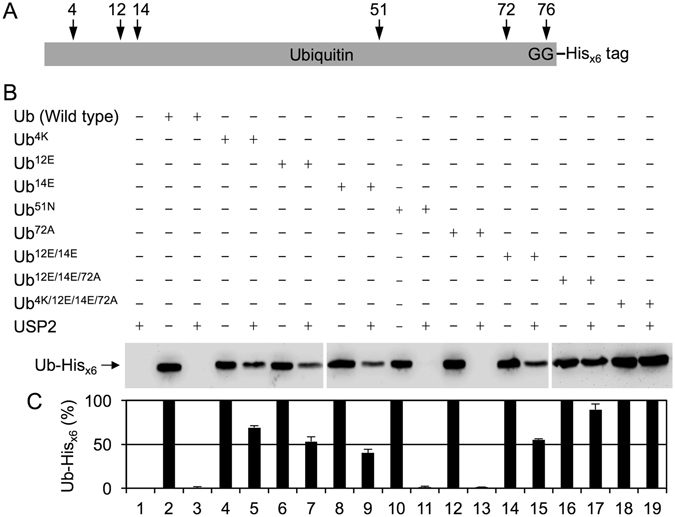
Residues at positions 4, 12, 14, and 72 of Ub are the molecular determinants for specific binding and catalysis by USP2. (**A**) A schematic diagram of the primary structure of the recombinant Ub used in the study, whose C-terminal di-glycine motif was fused with a His_x6_ tag (noted as Ub-His_x6_). USP2 cleaves on the normal peptide bond between the di-glycine motif (noted as GG) of Ub and the His_x6_ tag. The key residues of Ub interacting with USP2 were also labeled and marked with arrows. (**B**) Wild-type Ub-His_x6_ and indicated mutants (9.2 μM) were incubated with or without USP2 (4.75 μM) at 37 °C for 60 min. All reactions were terminated by adding 4X SDS-PAGE sample buffer and incubating at 100 °C for 10 min. Samples were separated on 16.6% SDS-PAGE and further analyzed using western blotting with the anti-His_x6_ tag antibody. Data are representative of three independent experiments. The full-length blots are presented in Supplementary Figure S1. (**C**) The signal intensity of three independent experiments was measured by a densitometer and processed by ImageJ. The results of the measurement were interpreted as a bar graph. Values are means ± S.D. from three independent experiments. The numbers noted at the bottom represent the lane numbers on the western blot.

Extensive evidence on the structural arrangement between the USP catalytic domain and the corresponding Ub molecule provided much useful information for experimental designs in the present study. The key residues of Ub, which are proposed to be involved in a specific interaction with USP2, are aligned with the equivalent residues of NEDD8 (Fig. 1). The N-terminal regions of Ub and NEDD8 contain three major residues, and the C-terminal 10 consecutive residues only have one residue difference between them. By using mutagenesis to replace the non-conserved residues 4, 12, 14, and 72 of Ub with their NEDD8 equivalents, and vice versa, a series of Ub and NEDD8 substrates with a C-terminal His_x6_ tag fused to their di-glycine motif were generated for analyzing the catalytic activity of USP2 (Fig. 2A). After wild-type Ub-His_x6_ was incubated with USP2 for 60 min, the C-terminal His_x6_ tag was completely removed and was not detected by using western blotting with anti-His_x6_ tag antibody due to the normal peptide bond between the di-glycine motif of Ub and the His_x6_ tag was cleaved by USP2 (Fig. 2B, lane 3). However, USP2 only partially cleaved Ub^4K^, Ub^12E^, and Ub^14E^ (Fig. 2B, lanes 5, 7 and 9), indicating that residues 4, 12, and 14 interacted with USP2. In addition, the C-terminal His_x6_ tags of Ub^51N^ and Ub^72A^ were completely removed by USP2 without showing any inhibition (Fig. 2B, lanes 11 and 13). It has been shown that residues 51 and 72 are the major determinants for specific recognition by the NEDD8-specific peptidase SENP8^32^. Thus, USP2 and SENP8 discriminate Ub and NEDD8 by recognizing different residues. Furthermore, USP2 cleaved Ub^12E/14E^ with a similar rate as it did Ub^12E^ or Ub^14E^ (Fig. 2B, lane 15), indicating that the binding of USP2 to these two sites is not purely additive. Moreover, Ub^12E/14E/72A^ was barely cleaved by USP2 (Fig. 2B, lane 17), indicating that the mutation of residue 72 might further decrease the interacting strength of interaction with USP. Residue 72 does still interact with USP2, but the strength or priority of the interaction is less than that of residues 12 and 14. Finally, we discovered that USP2 cannot cleave Ub^4K/12E/14E/72A^ (Fig. 2B, lane 19), suggesting that the N-terminal region of Ub is indeed crucial in its specific and strong binding with USP2 and USP2 might simultaneously recognize both of the N- and C-terminal key residues of Ub to secure the specific binding. To investigate whether the results are reproducible, three independent experiments were performed. The means and standard deviations were calculated and shown in Fig. 2C, which revealed the same manner as the results of Fig. 2B.

### Comparison of the reaction rates of various Ub substrates catalyzed by USP2

**Figure 3:**
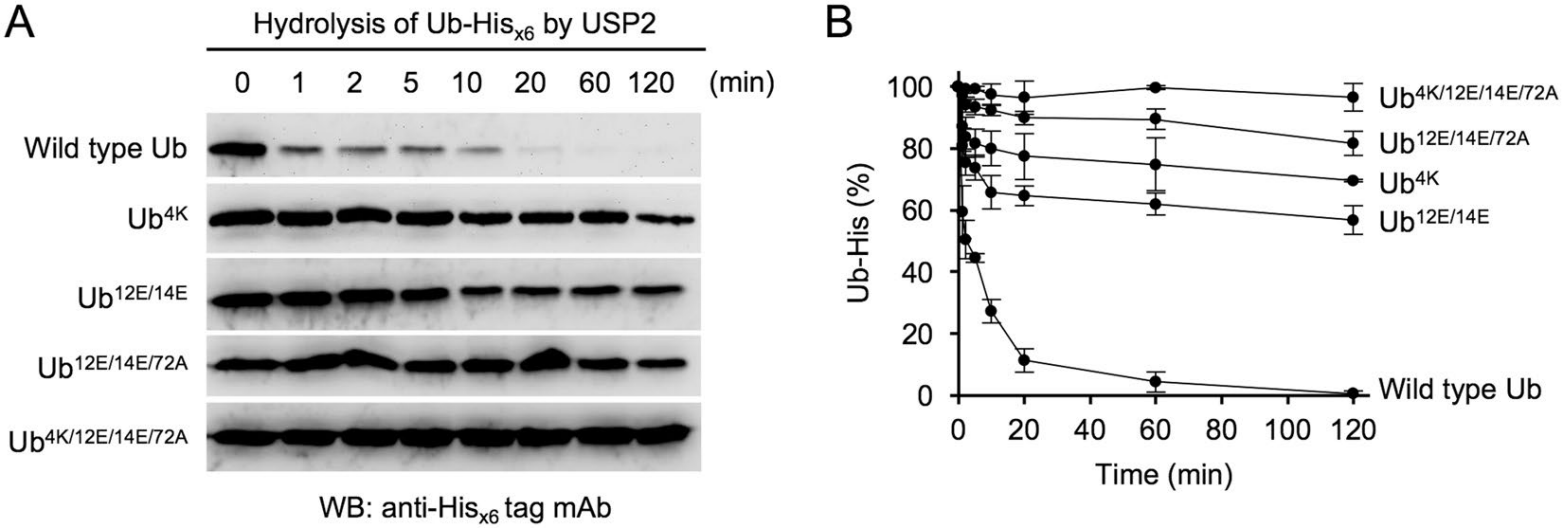
Comparison of the reaction kinetics of various Ub substrates catalyzed by USP2. (**A**) The Ub-His_x6_ mutants which can be cleaved by USP2 were selected to perform a time-course experiment. Wild-type Ub-His_x6_ and indicated mutants (9.2 μM) were incubated with USP2 (4.75 μM) at 37 °C for 1–120 min. At each time point, reactions were terminated by adding 4X SDS-PAGE sample buffer and incubating at 100 °C for 10 min. All of collected samples were separated on 16.6% SDS-PAGE and further analyzed using western blotting with the anti-His_x6_ tag antibody. Data are representative of three independent experiments. The full-length blots are presented in Supplementary Figure S2. (**B**) The signal intensity of three independent experiments was measured by a densitometer and processed by ImageJ. The results of the measurement were interpreted as a line chart. Values are means ± S.D. from three independent experiments.

To investigate the catalytic efficiency of USP2 toward Ub that was interrupted by the substitution of residues 4, 12, 14, and 72 of Ub with their NEDD8 equivalents, time-course experiments were used to examine the cleavage of the C-terminal His_x6_ tags from the substrates by USP2 during various incubation periods from 1 to 120 min. The majority of the C-terminal His_x6_ tag of wild-type Ub was efficiently removed by USP2 within 10 min (Fig. 3A) under the same assay conditions used for experiments detailed in Fig. 2. After 60 min of incubation, the C-terminal His_x6_ tag of wild-type Ub was completely removed by USP2. However, the reaction rates of Ub^4K^, Ub^12E/14E^, and Ub^12E/14E/72A^ were much lower than those of wild-type Ub. The replacement of Ub residue 4 with its NEDD8 equivalent efficiently prevented it from undergoing USP2 hydrolysis, indicating that the major sequence difference between Ub and NEDD8 at the N-terminus is a crucial determinant for specific substrate recognition. The degradation rate of Ub^12E/14E/72A^ was lower than that of Ub^12E/14E^, suggesting that residue 72, which is close to the C-terminal di-glycine motif at residues 75 and 76, accurately interacted with USP2 to control substrate accessibility to the catalytic site. As demonstrated previously, Ub^4K/12E/14E/72A^ completely inhibited its hydrolysis by USP2, revealing that these four residues are the molecular determinants for Ub-specific recognition by USP2. To investigate whether the results are reproducible, three independent time-course experiments were performed. The means and standard deviations were calculated and shown in Fig. 3B. The hydrolysis of wild-type Ub-His and indicated mutants by USP2 is linear within 10 min (Fig. 3B). The data obtained from the results of 10-min incubation time still clearly demonstrated that different single or multiple mutations at residues 4, 12, 14, and 72 of Ub-His changed the catalytic kinetics of USP2. Noted that hydrolysis of Ub^4K/12E/14E/72A^ mutant by USP2 is completely inhibited (Fig. 3B).

### NEDD8^4F/12T/14T/72R^ becomes a substrate of USP2

**Figure 4:**
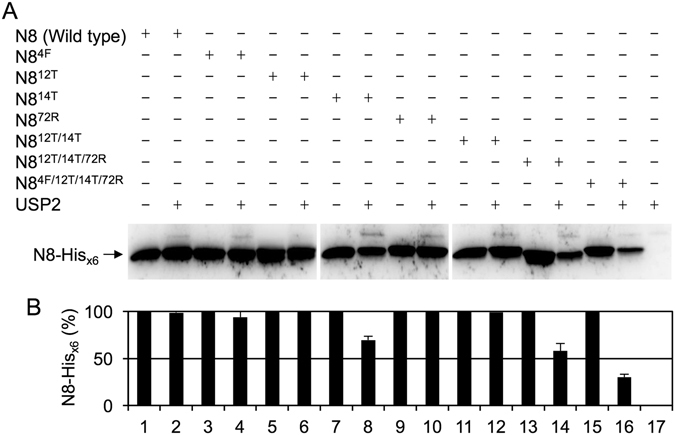
NEDD8 must simultaneously substitute the residues 4, 12, 14 and 72 with the Ub equivalents for efficient hydrolysis by USP2. (**A**) Wild-type NEDD8-His_x6_ and indicated mutants (13.8 μM) were incubated with or without USP2 (4.75 μM) at 37 °C for 60 min. All reactions were terminated by adding 4X SDS-PAGE sample buffer and incubating at 100 °C for 10 min. Samples were separated on 16.6% SDS-PAGE and further analyzed using western blotting with the anti-His_x6_ tag antibody. Data are representative of three independent experiments. The full-length blots are presented in Supplementary Figure S3. (**B**) The signal intensity of three independent experiments was measured by a densitometer and processed by ImageJ. The results of the measurement were interpreted as a bar graph. Values are means ± S.D. from three independent experiments. The numbers noted at the bottom represent the lane numbers on the western blot.

To determine whether the non-conserved residues 4, 12, 14, and 72 of NEDD8 also serves as the key determinants to prevent its hydrolysis by USP2, a series of NEDD8 mutants with a C-terminal His_x6_ tag fused to the di-glycine motif were generated and their reactivity was analyzed. NEDD8 was not hydrolyzed by USP2 (Fig. 4A, lane 2), whereas NEDD8^4F^ and NEDD8^14T^ were slightly accessible for processing by USP2 and revealed minor cleavage activity (Fig. 4A, lanes 4 and 8). Moreover, NEDD8^12T^, NEDD8^72R^, and NEDD8^12T/14T^ were not processed by USP2 (Fig. 4A, lanes 6, 10, and 12). Approximately half of the C-terminal His_x6_ tag of NEDD8^12T/14T/72R^ was removed by USP2 (Fig. 4A, lane 14), suggesting that the binding of USP2 to these two low-affinity sites (residues 12/14, and residue 72) was cooperative but not purely additive. Therefore, although residues 12 and 14 of NEDD8 were replaced with their Ub equivalents, an additional replacement of residue 72 of NEDD8 with its Ub equivalent led to more prominent USP2 processing activity. Furthermore, the experimental result revealed that NEDD8^4F/12T/14T/72R^ was efficiently processed by USP2 (Fig. 4A, lane 16), indicating that these four residues are indeed the molecular determinants for Ub-specific recognition by USP2. To investigate whether the results are reproducible, three independent experiments were performed. The means and standard deviations were calculated and shown in Fig. 4B, which revealed the same manner as the results of Fig. 4A.

### Comparison of the reaction rates of Ub and NEDD8^4F/12T/14T/72R^ catalyzed by USP2

**Figure 5:**
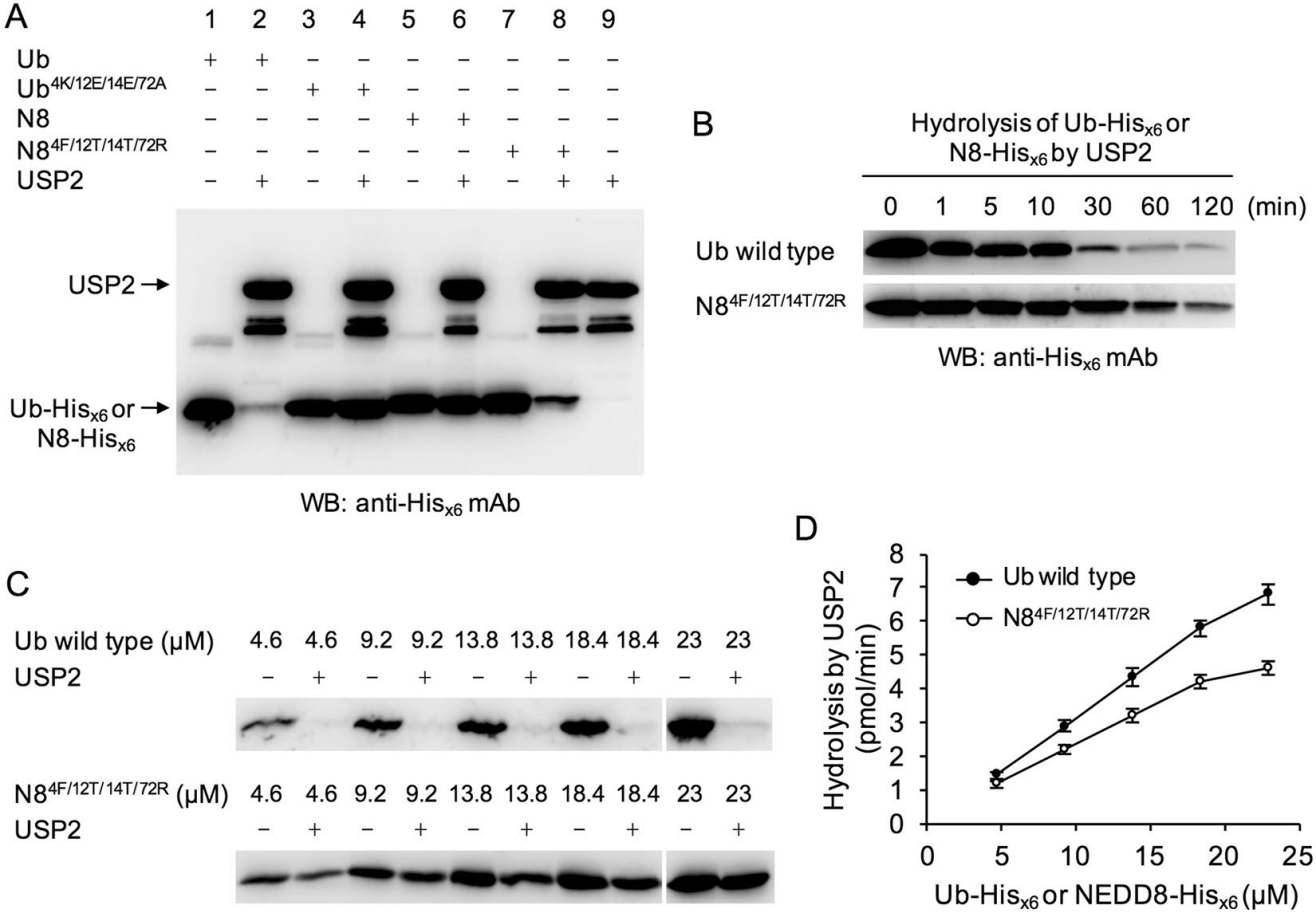
Comparison of the reaction kinetics of Ub and NEDD8^4F/12T/14T/72R^ catalyzed by USP2. (**A**) Ub, Ub^4K/12E/14E/72A^, NEDD8, and NEDD8^4F/12T/14T/72R^ (13.8 μM) were incubated with or without USP2 (4.75 μM) at 37 °C for 60 min. All reactions were terminated by adding 4X SDS-PAGE sample buffer and incubating at 100 °C for 10 min. Samples were separated on 16.6% SDS-PAGE and further analyzed using western blotting with the anti-His_x6_ tag antibody. (**B**) In order to measure the reaction kinetics of Ub and NEDD8^4F/12T/14T/72R^ catalyzed by USP2, Ub or NEDD8^4F/12T/14T/72R^ (13.8 μM) was incubated with USP2 (4.75 μM) to perform a time-course experiment for 120 min. At each time point, reactions were terminated by adding 4X SDS-PAGE sample buffer and incubating at 100 °C for 10 min. All of collected samples were separated on 16.6% SDS-PAGE and further analyzed using western blotting with the anti-His_x6_ tag antibody. The full-length blots are presented in Supplementary Figure S4. (**C**) To perform a standard curve calibration which can determine the linear reaction range, the various amounts of Ub-His_x6_ and NEDD8^4F/12T/14T/72R^-His_x6_ (4.6, 9.2, 13.8, 18.4, and 23 μM) were incubated with USP2 (4.75 μM) to conduct the same experimental protocol as described in (**A**). Data are representative of three independent experiments. The full-length blots are presented in Supplementary Figure S5. (**D**) The signal intensity of the unprocessed His-tagged substrates from three independent experiments was measured by a densitometer and processed by ImageJ. The reaction rates were calculated according to the His-tagged substrates which were hydrolyzed by USP2 and further plotted against the substrate concentrations used in the indicated reactions. Values are means ± S.D., n = 3.

To further confirm that the sequences at residues 4, 12, 14, and 72 of Ub and NEDD8 serve as the molecular determinants for specific substrate recognition, the hydrolysis of Ub, Ub^4K/12E/14E/72A^, NEDD8, and NEDD8^4F/12T/14T/72R^ by USP2 was measured in the same experiment. Ub^4K/12E/14E/72A^ and NEDD8 were not cleaved by USP2 (Fig. 5A, lanes 4 and 6), whereas Ub and NEDD8^4F/12T/14T/72R^ were efficiently cleaved by USP2 (Fig. 5A, lanes 2 and 8), indicating that the exchange of equivalent residues 4, 12, 14, and 72 between Ub and NEDD8 have consequently changed the specific recognition by USP2.

As revealed previously, the C-terminal His_x6_ tags of Ub and NEDD8^4F/12T/14T/72R^ were completely processed by USP2 after 60 min of incubation. To compare the reaction rates of Ub and NEDD8^4F/12T/14T/72R^ when catalyzed by USP2 over the entire 120 min of incubation, assays using the increased amount of Ub or NEDD8^4F/12T/14T/72R^ were applied to measure the hydrolysis of Ub or NEDD8^4F/12T/14T/72R^ by USP2. The results clearly demonstrated that USP2 still preferred to cleave Ub rather than NEDD8^4F/12T/14T/72R^ (Fig. 5B), implying some minor sequence differences other than residues 4, 12, 14, and 72 may also contribute to substrate recognition. Some structural differences between Ub and NEDD8 may affect their binding and cleavage by USP2.

To further confirm whether the USP2 enzymatic reactions were analyzed and measured within a linear range, the increasing amounts of Ub-His_x6_ and NEDD8^4F/12T/14T/72R^-His_x6_ (4.6–23 μM) were incubated with USP2 to perform a standard curve calibration by conducting the experiments with the same assay protocol as described previously (Fig. 5C). The data showed that the enzymatic reactions were linear within the experimental conditions using 4.6–18.4 μM of Ub-His_x6_ and NEDD8^4F/12T/14T/72R^-His_x6_ as the substrates (Fig. 5D). The results supported that the hydrolysis of 9.2 μM or 13.8 μM of Ub-His_x6_ and NEDD8^4F/12T/14T/72R^-His_x6_ by USP2 analyzed in Figs 2, 3, 4 and 5 were measured within a linear range.

### USP21 displayed the same substrate recognition mechanism as USP2

**Figure 6:**
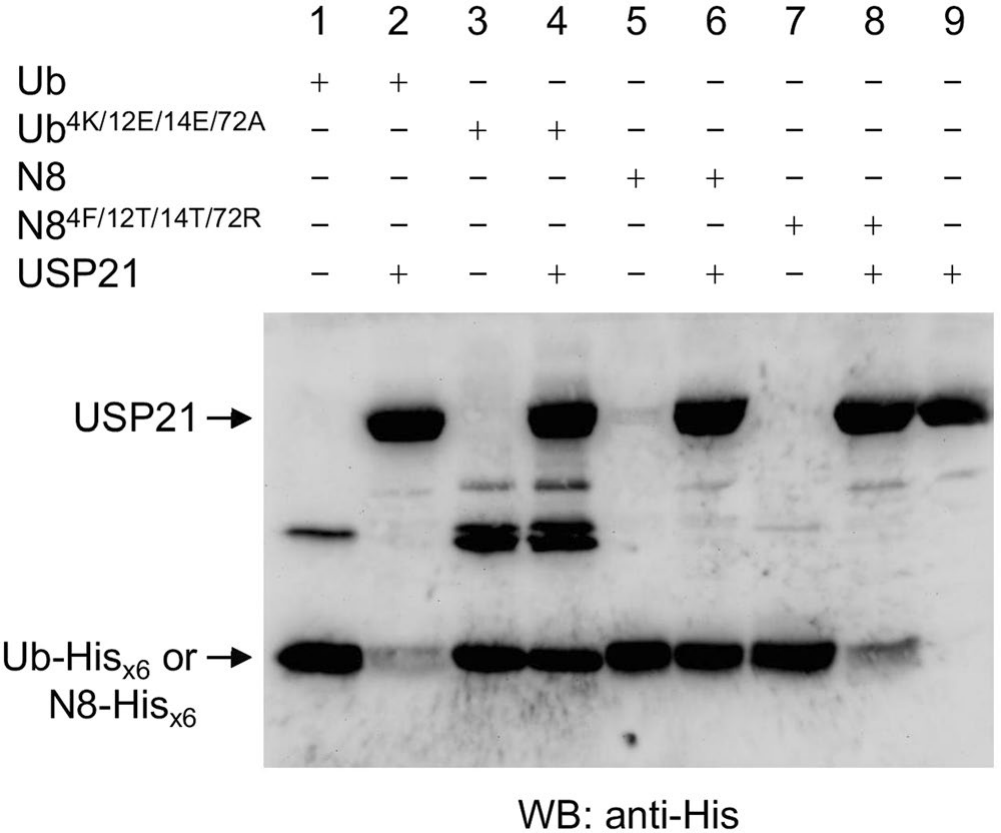
USP21 displayed the same substrate recognition mechanism as USP2. Ub, Ub^4K/12E/14E/72A^, NEDD8, and NEDD8^4F/12T/14T/72R^ (13.8 μM) were incubated with or without USP21 (4.425 μM) at 37 °C for 60 min. Samples were separated on 16.6% SDS-PAGE and further analyzed using western blotting with the anti-His_x6_ tag antibody.

The USP protein family contain a conserved catalytic domain. Thus, we also determined whether USP21 has the same substrate recognition mechanism as USP2. The data revealed that, under the same assay conditions, USP21 did not cleave Ub^4K/12E/14E/72A^ or NEDD8 (Fig. 6, lanes 4 and 6) but almost completely cleaved Ub and NEDD8^4F/12T/14T/72R^ (Fig. 6, lanes 2 and 8), indicating that USP21 has the same substrate specificity toward Ub as USP2 through recognition of residues 4, 12, 14, and 72.

## Discussion

**Figure 7:**
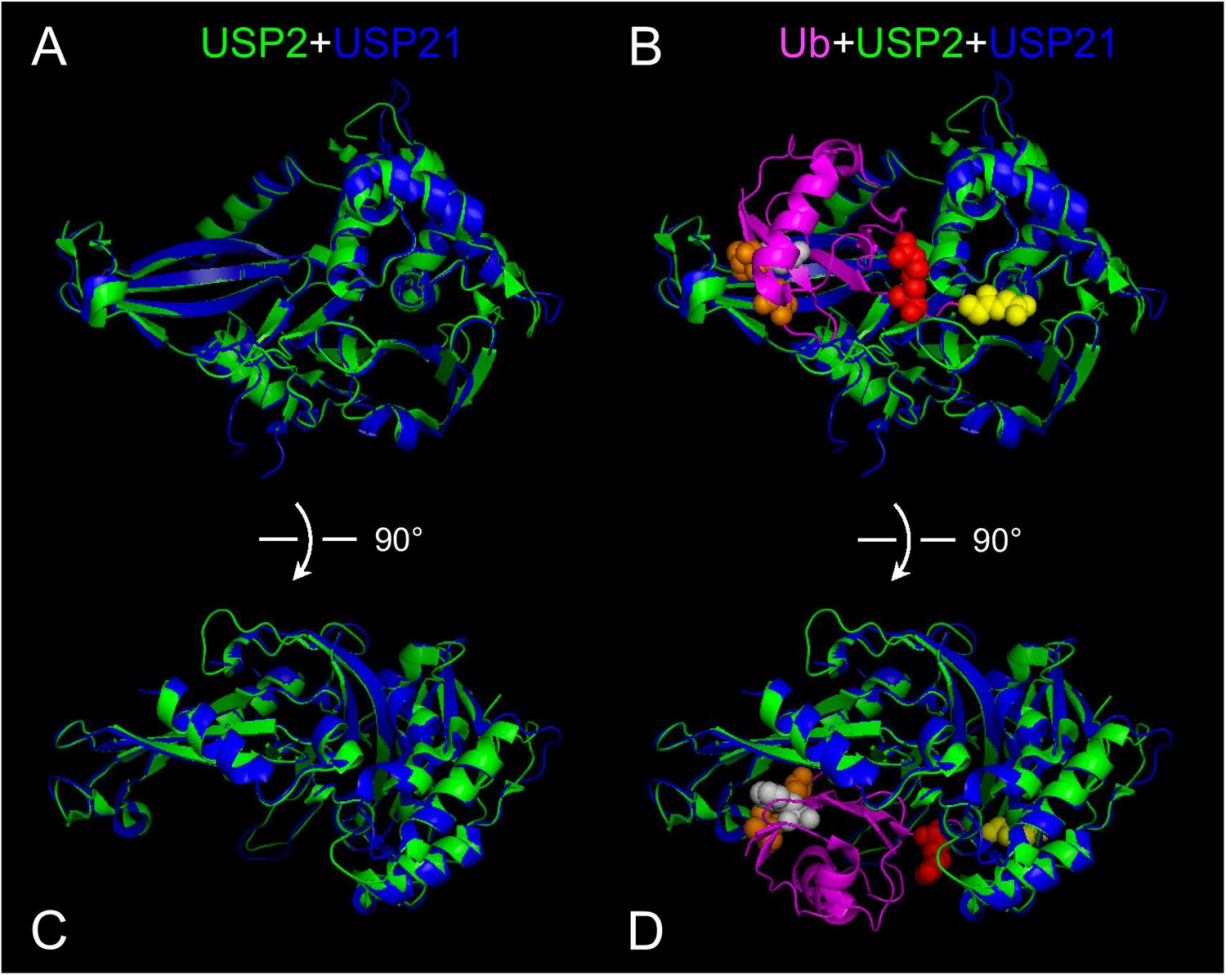
The superposition of USP2 with USP21. (**A**) The 3-D structure of USP2-core (PDB ID: 2HD5) in green was superimposed with the 3-D structure of USP21 (PDB ID: 3MTN) in blue by using PyMOL software. The overall structure resembles a cupped hand comprising a conserved Fingers-Palm-Thumb architecture. (**B**) The Ub-based suicide probe (shown in magenta) was added to the superimposed images of USP2-core and USP21-core. The C-terminal di-glycine motif was shown in yellow, Arg-72 was shown in red, Thr-12 and Thr-14 were shown in orange, and Phe-4 was shown in grey. For better recognition of the indicated residues, they were also noted as spheres. (**C** and **D**) The superimposed images of (**A** and **B**) were rotated 90 degree along with the x-axis to reveal another view angle and the close interaction between the N-terminal region of Ub and the tip of the Fingers of USP2.

Human Ub and NEDD8 share 58% sequence identity, 80% sequence similarity, and have a highly conserved three-dimensional (3-D) structure. Among the last ten C-terminal residues of Ub and NEDD8, the only different residue is the one at position 72. The modification of target proteins by Ub and NEDD8 may have distinct biological consequences^1, 2, 9–12^. Therefore, it is necessary to study how USPs can distinguish Ub from NEDD8 and thus maintain the unique substrate specificity. However, despite limited sequence identity among the large USP protein family, USPs have a conserved catalytic domain. For instance, the N-terminal fragments of USP2 and USP21 share only 27% sequence identity, but their C-terminal catalytic domains (USP2-core and USP21-core) share 50% sequence identity, suggesting that the catalytic domains of USPs are the major components responsible for recognizing Ub. In fact, USP2-core and USP21-core, composed of three structural elements (Fingers, Palm, and Thumb), superimpose especially well with an RMSD of 0.687 Å for 280 aligned Cα atoms (Fig. 7A and C). This structural conservation ensures the specificity of Ub binding by using their highly conserved determinants in the USP catalytic domain.

On the basis of the structural data of the USP2-Ub complex (Fig. 7B) and the sequence alignment of Ub and NEDD8 (Fig. 1), the major molecular determinants for specific Ub NEDD8 discrimination by USP2 are probably located at the N-terminus and the C-terminus of Ub, which interact with the tip of the Fingers, and with the Palm and Thumb of USP2, respectively. Thus, we examined whether the non-conserved residues 4, 12, 14, and 72 play crucial roles in specific binding with USP2. Our data demonstrated that a single substitution of the N-terminal residue 4, 12, or 14 of Ub with the equivalent residue of NEDD8 may lead to strong inhibition of hydrolysis by USP2 (Fig. 2B, lanes 5, 7, and 9). Surprisingly, a single replacement of Ub residue 72 with its equivalent NEDD8 residue did not result in any inhibition (Fig. 2B, lane 13), suggesting that binding of the C-terminus of Ub to USP2 was not adequately stable. However, the hydrolysis of Ub^12E/14E/72A^ by USP2 was substantially lower than the hydrolysis of Ub^12E/14E^, implying that once the strong binding between the N-terminus of Ub and USP2 had occurred, residue 72 and the C-terminus peptide strengthened the binding and further aided the alignment of the C-terminal di-glycine motif to reach for the active site. This model also concluded that USP2 bound with the N-terminus of Ub to form a stable interaction and then bound to the C-terminus of Ub to ensure substrate specificity. It has been reported that the catalytic mechanism of the HAUSP catalytic core in complex with Ub resembles the induced-fit binding model. Binding of Ub triggers a drastic conformational change in the active site of HAUSP that realigns the catalytic triad for specific catalysis on the C-terminal di-glycine motif of Ub^36^. In the present study, we only measured the catalytic activity of USP2 against Ub and Ub^R72A^ without characterizing the structural features of USP2 in complex with Ub and Ub^R72A^ by X-ray diffraction. Therefore, we cannot come to a conclusion that the residue 72 of Ub may trigger a conformational change in the catalytic site of USP2.

More evidence supporting this Ub and USP2 binding model was revealed from the results of the hydrolysis of NEDD8 mutants by USP2. The single replacement of the residues 4, 12, and 72 of NEDD8 with their Ub equivalent residues did not result in any hydrolysis by USP2 (Fig. 4A, lanes 4, 6, and 10). Only NEDD8^12T/14T/72R^ and NEDD8^4F/12T/14T/72R^ were cleaved efficiently by USP2 (Fig. 4A, lanes 14 and 16), suggesting that both the N- and C-terminal sites of the Ubls must bind correctly and simultaneously if specific recognition and catalysis by USP2 is to be achieved. Additionally, USP21 was used to investigate whether other USPs use the same mechanism to distinguish between Ub and NEDD8. As illustrated in Fig. 6, USP21 had the same substrate specificity due to correctly identifying residues 4, 12, 14, and 72 on Ub and NEDD8, suggesting that the conserved structure of USPs does indeed enable them to distinguish minor differences between Ub and NEDD8. Notably, Ye *et al*. reported that USP21 can react with an NEDD8^4F/12T/14T/72R^-C2Cl suicide probe but not with an NEDD8-C2Cl suicide probe^39^. We also observed that USP21 could cleave the C-terminal His_x6_ tag of NEDD8^4F/12T/14T/72R^ mutant but not that of the wild-type NEDD8 by using a different experimental method (Fig. 6). However, Gong *et al*. have reported that USP21 can have dual specificity for Ub- and NEDD8-conjugated proteins^26^. These studies appear to have obtained contradictory results, but we posit that these differences may have arisen from the different biochemical analyses used with either the catalytic core or the full length of USP21 and various forms of substrates employed.

In the present study, the catalytic activity of USP was investigated using a Ub substrate with a His_x6_-tag fused at the C-terminus of the di-glycine motif. This method was different from those employed in previous studies, which used a potent Ub-based suicide probe such as Ubal^35^ or Ub-C2Cl^42^ to examine the covalent modification at the active site of USP. Nonetheless, our method can directly measure the catalytic reaction of USP2 through detecting cleavage of the normal peptide bond between the di-glycine motif of Ub and the C-terminal His_x6_ tag. This method does not measure the cleavage of the complex isopeptide bonds formed in the hyper-neddylated substrates. With this experimental design, the kinetic study of the catalytic rate and the efficiency of USP toward different substrates was possible. The results of time-course experiments (Fig. 3) demonstrated that different single or multiple mutations at residues 4, 12, 14, and 72 of Ub changed the catalytic kinetics of USP2. Moreover, the inhibition caused by two or more mutations was not purely additive when compared with those observed when single mutations were made (Fig. 3B). In conclusion, our data revealed that binding of the N-terminus of Ub to the Fingers of USP2 may occur first for forming a stable interaction. The C-terminus of Ub was then aligned into a narrow channel between the Palm and the Thumb regions, after which catalysis occurs. The substrate of USP2 must simultaneously contain the correct residues 4, 12, 14 and 72 at the N- and C-termini if it is to be specifically recognized by USP2.

## Methods

### Cell culture

HeLa cells were grown in Dulbecco’s modified Eagle’s medium (Gibco, Thermo Fisher Scientific) supplemented with 10% fetal bovine serum (Gibco, Thermo Fisher Scientific) under optimum growth conditions (37 °C, 5% CO_2_).

### Total mRNA isolation and reverse transcription

The total mRNA of HeLa cells was obtained using FastTrack 2.0 mRNA Isolation Kit (Invitrogen, Thermo Fisher Scientific) according to the manufacturer’s instruction. The reverse transcription of the first-strand cDNA was performed at 37 °C for 50 min using M-MLV (Moloney Murine Leukemia Virus) Reverse Transcriptase (Invitrogen, Thermo Fisher Scientific) according to the manufacturer’s instruction.

### Primer design, cloning and plasmid construction

The cDNAs encoding for human Ub and NEDD8 were amplified by standard PCR method using the Phusion High-Fidelity PCR Kit (Thermo Fisher Scientific) with the following primer sets: Ub-forward, 5′-GATGGATCCATGCAGATCTTCGTCAAGACGTTAACCGGTAAA-3′ (BamHI site underlined); Ub-reverse,5′-CAGGCGGCCGCACCACCTCTTAGTCTTAAGAC-3′ (NotI site underlined); NEDD8-forward, 5′-GAT GGATCC ATGCTAATTAAAGTGAAGACGCTGACCGG-3′ (BamHI site underlined); NEDD8-reverse, 5′-ACT GCGGCCGC TCCTCCTCTCAGAGCCAACACCAGGTGAAGGA-3′ (NotI site underlined). For constructing the HA-Ub-His_x6_ and HA-NEDD8-His_x6_ expression plasmids, the cDNA of Ub or NEDD8 flanked with BamHI and NotI cutting sites was cloned into the modified pET-28a-HA plasmid (Novagen, EMD Millipore) without a stop codon for fusing with a His_x6_-tag coding sequence. For constructing the expression plasmid of the catalytic domain of human USP21 (a gift from Dr. Wade Harper, Addgene plasmid #22574), the cDNA encoding for residues Ala201 to Glu560 was amplified by standard PCR method using the Phusion High-Fidelity PCR Kit with the following primer sets: forward, 5′-AATGAATTCATGGCTCATCACACACTCCTTCT-3′ (EcoRI site underlined); reverse, 5′-CGCTCGAGCTCCTGCATCAGTTGGTAGAACAGC-3′ (XhoI site underlined), and subcloned into pET28a vector. The sequences of the expression plasmids were further verified by DNA sequencing.

### Site-directed mutagenesis

All Ub and NEDD8 mutants were generated by the PCR-based QuikChange II Site-Directed Mutagenesis Kit (Stratagene, Agilent Technologies) according to the manufacturer’s instruction. All mutations were confirmed by DNA sequencing.

### Protein expression and purification

Expression vectors containing Ub, NEDD8, His-USP2-core (Met258 to Met605) (a gift from Dr. Daniel Taillandier^45^), and USP21 catalytic core were transformed into *E. coli* BL21(DE3) or Rosetta(DE3) cells (Novagen, EMD Millipore) individually. Cells were incubated at 37 °C on an orbital shaker at 180 rpm. Expression of the recombinant protein was induced at an A_600_ of 0.6–0.7 by adding isopropyl-1-thio-β-D-galactopyranoside to a final concentration of 1 mM for 4 h. His-tagged proteins were purified using HisTrap FF column (GE Healthcare Life Sciences) and bound proteins were eluted with a 20–500 mM gradient of imidazole in a buffer containing 20 mM NaH_2_PO_4_, pH 7.4 and 500 mM NaCl. The protein purity was examined by 16.6% SDS-PAGE and the concentration was determined by the Bradford dye-binding method^46^.

### Peptidase activity assay

To measure the USP activity, Ub-His or NEDD8-His was incubated with the purified USP2-core (4.75 μM) or USP21-core (4.425 μM) at 37 °C in buffer containing 50 mM Tris-HCl, pH 7.4, 50 mM NaCl and 5 mM β-mercaptoethanol. All reactions were terminated by adding 4X SDS-PAGE sample buffer and incubating at 100 °C for 10 min. Samples were separated on 16.6% SDS-PAGE followed by Coomassie Brilliant Blue R-250 staining, or blotted onto the PVDF membrane (EMD Millipore) and analyzed by western blotting using the anti-His antibody (GE Healthcare Life Sciences).

## Electronic supplementary material

Supplementary information
