# Supplementary material for: The molecular determinants for distinguishing between ubiquitin and NEDD8 by USP2
Source: Sci Rep. 2017 May 23;7:2304. doi: 10.1038/s41598-017-02322-x (PMC5442100; doi:10.1038/s41598-017-02322-x)
Supplement: Supplementary file 1 — Supplementary information [file 41598_2017_2322_MOESM1_ESM.pdf]

Supplementary information

The molecular determinants for distinguishing between ubiquitin and NEDD8 by USP2

Yung-Cheng Shin[1], Jou-Han Chen[1] and Shih-Chung Chang[1,2,*]

[1]Department of Biochemical Science and Technology, College of Life Science, National Taiwan University, Taipei, Taiwan.

[2]Center of Biotechnology, National Taiwan University, Taipei, Taiwan.

*Correspondence and requests for materials should be addressed to S.-C.C. (email: shihchung@ntu.edu.tw)

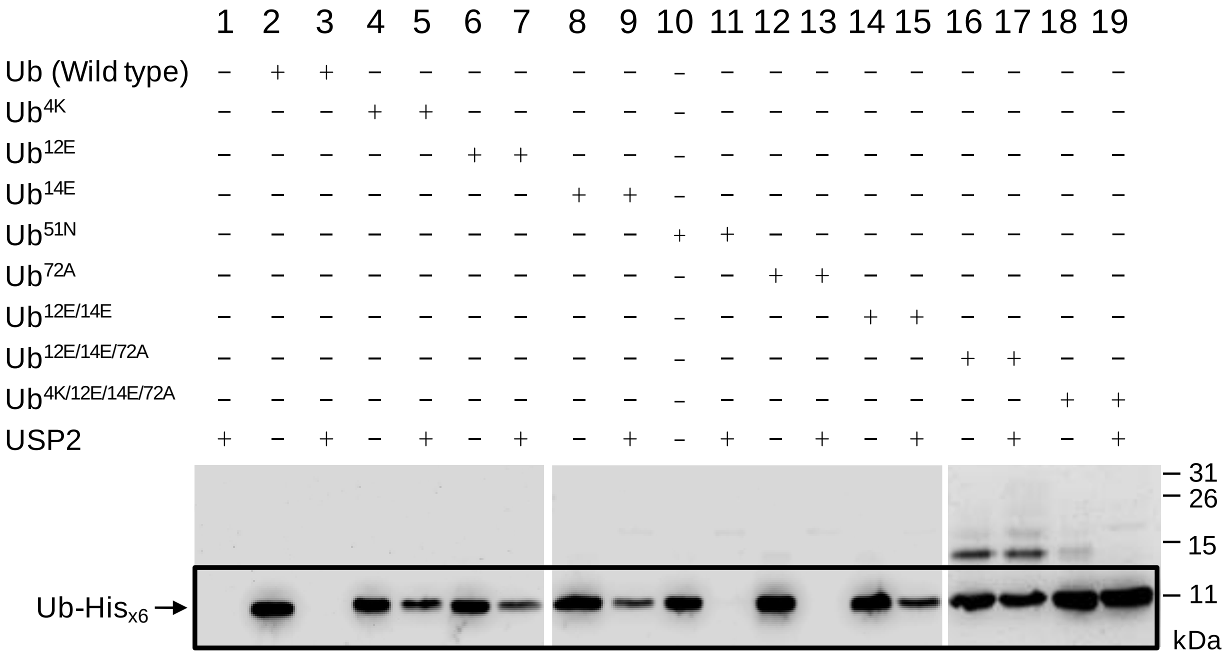

Supplementary Figure S1. Wild-type Ub-His$_{x6}$ and indicated mutants (9.2 μM) were incubated with or without USP2 (4.75 μM) at 37°C for 60 min. All reactions were terminated by adding 4X SDS-PAGE sample buffer and incubating at 100°C for 10 min. Samples were separated on 16.6% SDS-PAGE and further analyzed using western blotting with the anti-His$_{x6}$ tag antibody. The bands derived from full-length western blots used in the main text were framed as shown in the figure. Lanes 1–7 were cropped from one gel, lanes 8–15 were cropped from one gel, and lanes 16–19 were cropped from another gel because of the limited loading spaces on the same gel.

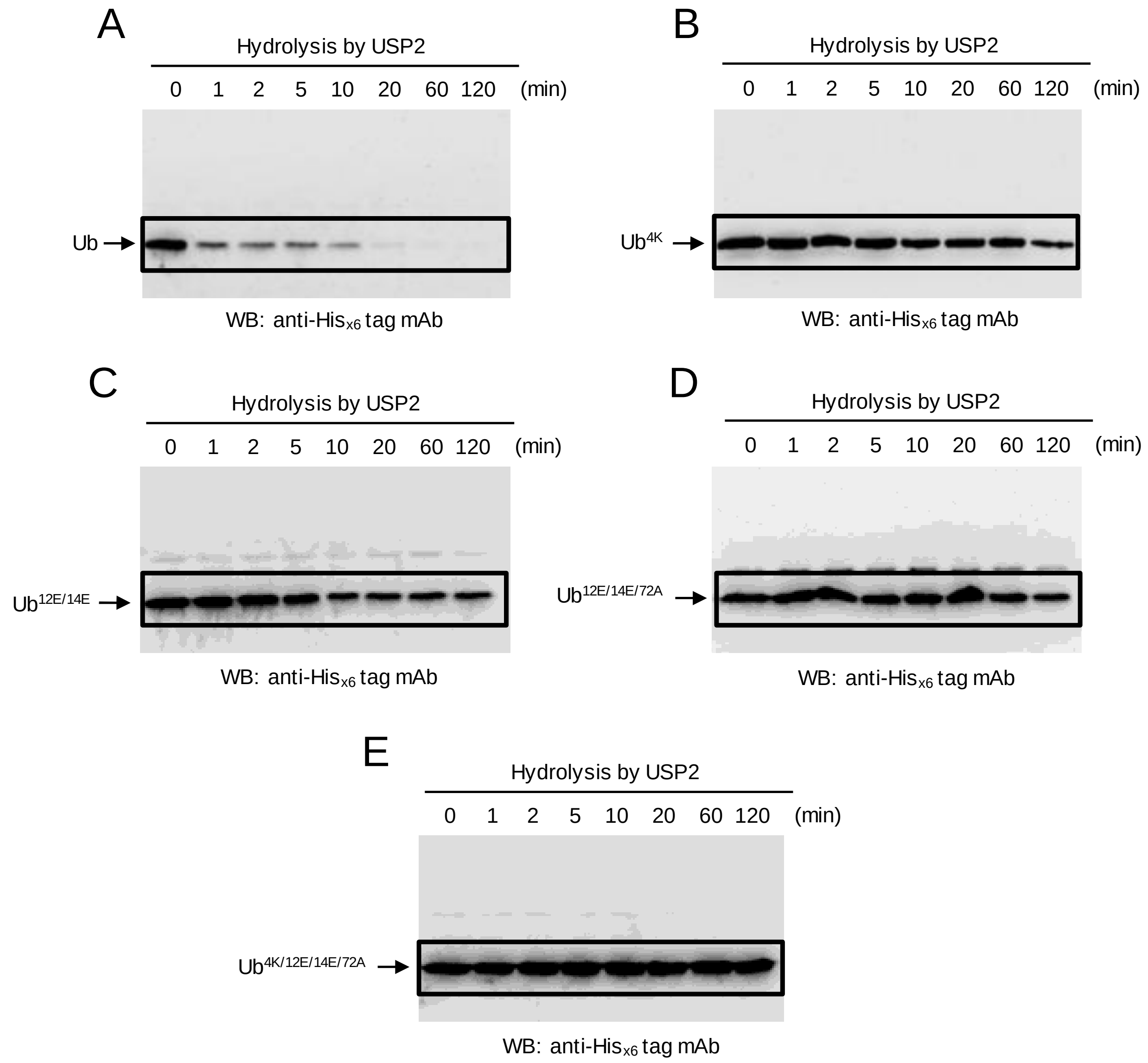

Supplementary Figure S2. Ub-His$_{x6}$ and Ub-His$_{x6}$ mutants (Ub^{4K}, Ub$^{12E/14E}$, Ub$^{12E/14E/72A}$, Ub$^{4K/12E/14E/72A}$) were selected to perform a time-course experiment. Ub-His$_{x6}$ (**A**) and indicated mutants (**B–E**) (9.2 μM) were incubated with USP2 (4.75 μM) at 37°C for 1–120 min. At each time point, reactions were terminated by adding 4X SDS-PAGE sample buffer and incubating at 100°C for 10 min. All of collected samples were separated on 16.6% SDS-PAGE and further analyzed using western blotting with the anti-His$_{x6}$ tag antibody. Data are representative of three independent experiments. The bands derived from full-length western blots used in the main text were framed as shown in the figure.

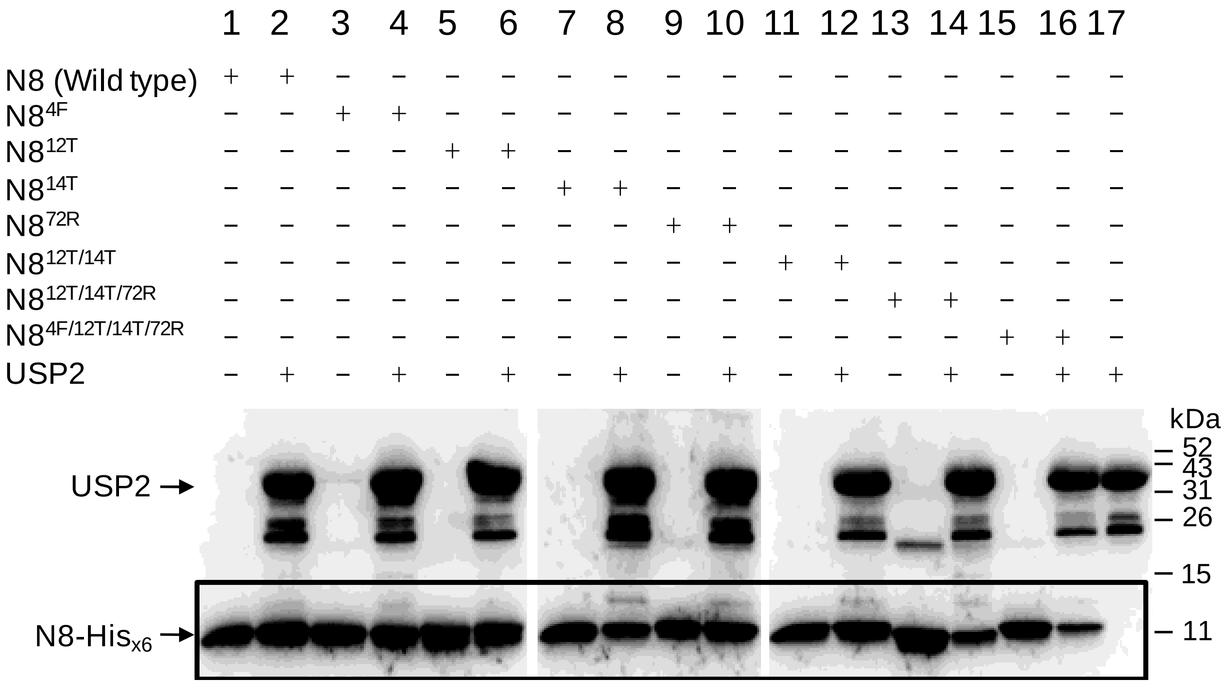

Supplementary Figure S3. Wild-type NEDD8-His$_{x6}$ and indicated mutants (13.8 μM) were incubated with or without USP2 (4.75 μM) at 37°C for 60 min. All reactions were terminated by adding 4X SDS-PAGE sample buffer and incubating at 100°C for 10 min. Samples were separated on 16.6% SDS-PAGE and further analyzed using western blotting with the anti-His$_{x6}$ tag antibody. The bands derived from full-length western blots used in the main text were framed as shown in the figure. Lanes 1–6 were cropped from one gel, lanes 7–10 were cropped from one gel, and lanes 11–17 were cropped from another gel because of the limited loading spaces on the same gel.

A

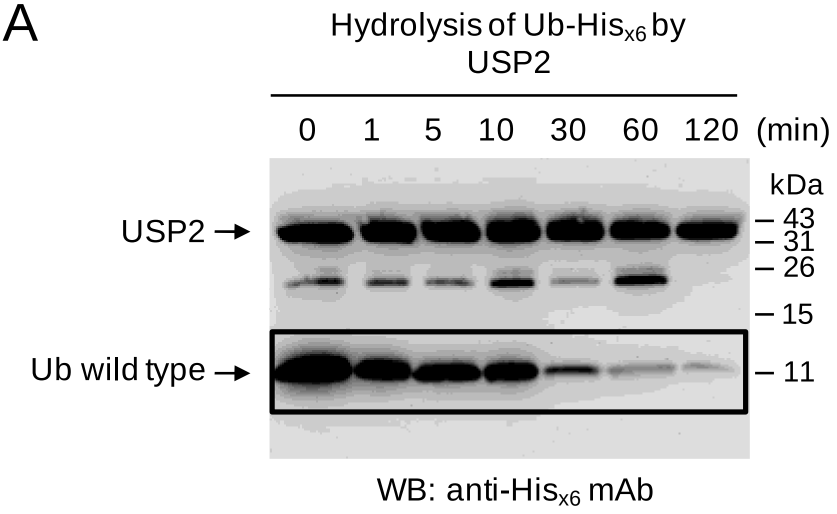

B

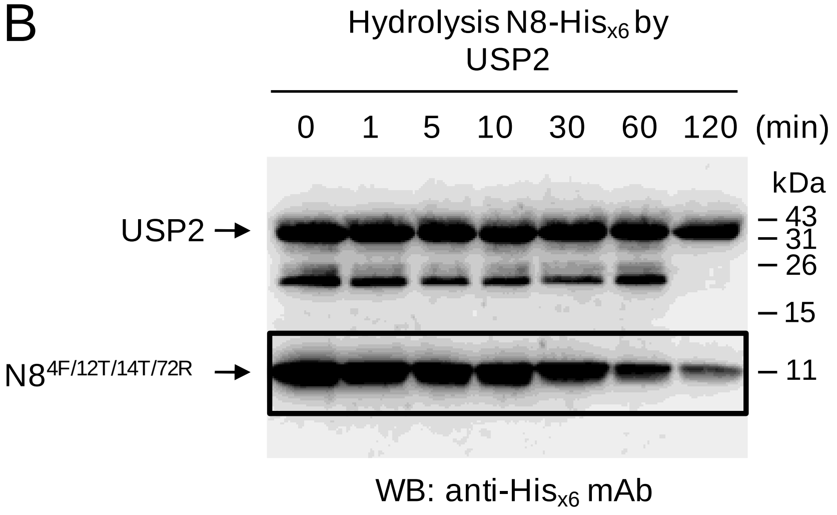

Supplementary Figure S4. Ub wild type (**A**) or NEDD8$^{4F/12T/14T/72R}$ (**B**) (13.8 μM) was incubated with USP2 (4.75 μM) to perform a time-course experiment for 120 min. At each time point, reactions were terminated by adding 4X SDS-PAGE sample buffer and incubating at 100°C for 10 min. All of collected samples were separated on 16.6% SDS-PAGE and further analyzed using western blotting with the anti-His$_{x6}$ tag antibody. The bands derived from full-length western blots used in the main text were framed as shown in the figure.

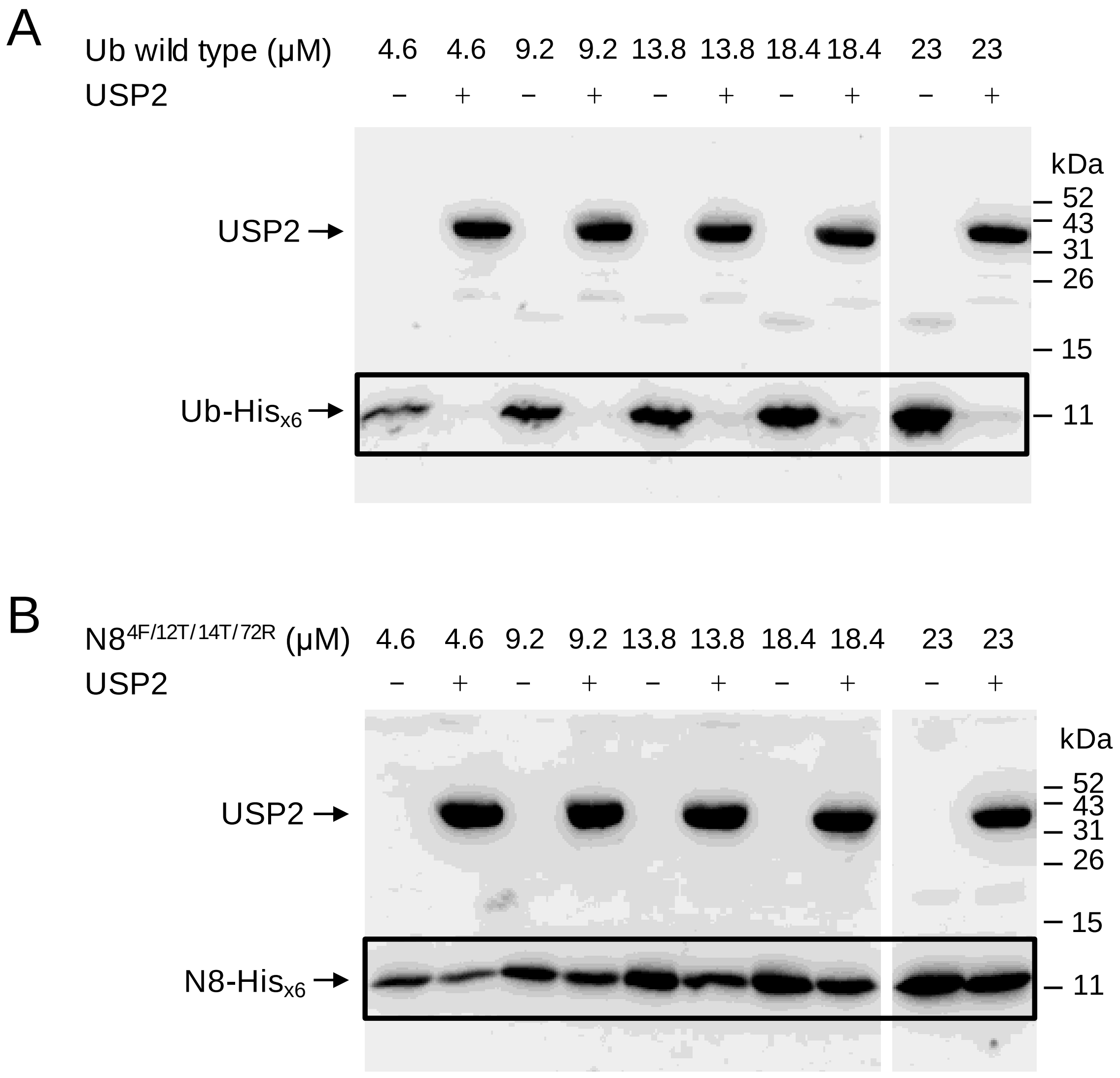

Supplementary Figure S5. The various amounts of Ub-His$_{x6}$ (**A**) and NEDD8$^{4F/12T/14T/72R}$-His$_{x6}$ (**B**) (4.6, 9.2, 13.8, 18.4, and 23 μM) were incubated with or without USP2 (4.75 μM) at 37°C for 60 min. All reactions were terminated by adding 4X SDS-PAGE sample buffer and incubating at 100°C for 10 min. Samples were separated on 16.6% SDS-PAGE and further analyzed using western blotting with the anti-His$_{x6}$ tag antibody. The bands derived from full-length western blots used in the main text were framed as shown in the figure. Lanes 1–8 (4.6, 9.2, 13.8, and 18.4 μM) were cropped from one gel, and lanes 9&10 (23 μM) were cropped from another gel because of the limited loading spaces on the same gel.
